# Behçet’s disease in Wales: an epidemiological description of national surveillance data

**DOI:** 10.1186/s13023-022-02505-4

**Published:** 2022-09-06

**Authors:** Annie Ashman, David Tucker, Ceri Williams, Llion Davies

**Affiliations:** 1 Public Health Wales, Capital Quarter 2, Tyndall Street, Cardiff, CF10 4BZ UK; 2 Congenital Anomaly Register and Information Service, Public Health Wales, Capital Quarter 2, Tyndall Street, Cardiff, CF10 4BZ UK

**Keywords:** Behçet’s disease, Rare disease, Adult disease, Wales, Surveillance registry

## Abstract

**Objectives:**

Behçet’s disease is a rare, chronic, incurable, multisystemic disease. It causes significant morbidity, with patients experiencing symptoms including mucous membrane ulcers, and joint pain and swelling. It is an important cause of avoidable blindness due to ocular involvement. The aetiology is unknown. The aims were to identify population prevalence of Behçet’s disease in Wales in comparison to other endemic and non-endemic regions, and provide an epidemiological profile of a case series of adult patients. This is the first analysis of data from the Adult Rare Diseases Surveillance Registry for Wales, established in 2020 as part of the COVID-19 pandemic response.

**Results:**

Between 1995 and 2020, 347 adults and 5 children were recorded in Wales with a diagnosis of Behçet’s disease. Population prevalence was calculated as 11.1 per 100,000 population. Of the adult cases, 76.9% were female, and 6.6% died before the end of the study period. When comparing genders, there were no statistically significant differences in age at diagnosis, mortality or socioeconomic status. There was no evidence that the age at which cases were diagnosed had changed over time. Survival analyses showed no significant differences in durations of survival between genders or individuals residing in different WIMD 2019 quintiles. Age at diagnosis was the only factor significantly and independently associated with poorer durations of survival (p < 0.001).

## Introduction

Orphanet describes Behçet’s disease as a “rare, chronic, relapsing, multisystemic vasculitis” [1]. Symptoms include mucous membrane lesions, such as mouth ulcers, genital ulcers and digestive tract ulcers that tend to be intermittent. Inflammation of the eyes may also occur [2]. Around half of cases suffer joint pain and swelling, which may become chronic, and up to 20 per cent will have involvement of the central nervous system [2]. Complications include blindness [3] and stroke [4], and studies have shown that symptoms tend to be more severe in younger patients [5]. Furthermore, most cases should have a normal lifespan despite the considerable morbidity associated with the disease [6]. The exact cause of Behçet’s disease is unknown, and the condition is incurable [1].

The prevalence of Behçet’s disease varies geographically, with the condition being most common along the ancient “Silk Road” route in the Far East and Mediterranean basin [3]. Reported prevalence has found to be as high as > 1 case per 1000 population in Turkey [1]. Orphanet reports a mean onset age of 30 years [1].

Developing strategies to support individuals with rare diseases (those with incidence of < 1 in 2000, and a code assigned in the Orphanet directory) is a current priority for the four UK nations, following publication of the Rare Diseases Framework. The framework aims include helping patients get a faster diagnosis, increasing awareness of rare diseases among healthcare professionals, better coordination of care, and improving access to specialist treatment, care and drugs [7].

This study aimed to identify the population prevalence of Behçet’s disease in Wales, and to provide an epidemiological description of a case series of adult patients recorded in the national surveillance registry.

## Methods

An adult rare diseases surveillance registry was established in Wales in June 2020 as part of the COVID-19 pandemic response. The initial focus was to collect data on the conditions included on the clinically extremely vulnerable (shielding) list, including Behçet’s disease.

Identification of Behçet’s disease cases for registration was a dual process. Firstly, anonymised data were transferred by registry staff from Public Health Wales’s Congenital Anomalies Register and Information Service (CARIS) for individuals now over 18 years of age. Secondly, data were accessed from the Patient Case Episodes Database Wales (PEDW). This secondary care database was implemented in 1991 and holds data on all emergency and planned hospital in-patient admissions in Wales. Demographic data were cross-referenced with the Welsh Demographic Service (WDS, a used by NHS services to identify cases and their addresses) to reconcile case data and address missing variables. As WDS records deaths, checks could be made on whether cases were still alive. As many rare disease patients have multiple contacts with the NHS, it was possible to check consistency of information across multiple admissions. Clinical portals were accessed, containing information including clinical letters, discharge summaries, referral letters and microbiological and radiological test results. This was to ensure that coding in PEDW was correct and to validate diagnoses, with escalation to a clinician if required, for example by manually checking for the most recent diagnosis where individuals had received previous misdiagnoses. Initial misdiagnosis or consideration of differential diagnoses is a common occurrence for patients with rare diseases as diagnosis can be challenging. To calculate population-level prevalence, paediatric cases of Behçet’s disease were also identified from the CARIS database.

The first step in handling data was to manually de-duplicate the records, acknowledging the potential for cases to be recorded more than once due to data being gathered from multiple datasets. Cases were then removed where diagnosis of Behçet’s disease was suspected, but not verified in the clinical records.

In order to adjust for deprivation, a new variable, the Welsh Index of Multiple Deprivation (WIMD) quintile of residence for each individual, was created. WIMD is the Welsh Government’s official measure of relative deprivation for small areas in Wales [8]. Using a postcode to WIMD rank look-up tool [9], a deprivation quintile between 1 and 5, with 1 being the most deprived, was inputted for each record.

To facilitate survival analysis, another new variable, survival in months from diagnosis, was created. Date of death was used as the censor point for those cases known to have died. Living cases had survival measured in months from date of diagnosis (the earliest mention of a coded episode) to the end of the study period.

Population prevalence was calculated by combining paediatric cases of Behçet’s disease recorded by CARIS, and living adult cases from the adult rare disease registry as the numerator. The denominator was the population of Wales as per the most recent Office for National Statistics mid-year population estimate at the study end date [10]. For the purposes of the epidemiological description, only adult cases were included.

The data were exported to SPSS (version 24.0, IBM, Chicago, IL) [11] for statistical analyses. Continuous data were summarised as median (range) and non-parametric tests used. Categorical and continuous data were compared with the Chi-square and Mann–Whitney U-tests respectively. Spearman’s Rho was used to investigate the relationship between date of diagnosis and age at diagnosis, to ascertain whether age at diagnosis had varied over time. The significance value was set at 5% (p < 0.05).

Univariable and multivariable survival analyses were conducted using Kaplan–Meier and Cox Regression (backward likelihood ratio) respectively. As the proportion of deaths across the cohort was relatively low (6.6%), formal median survival according to survival analyses were not possible, however survival was calculated manually using survival from date in diagnosis in months. The variables entered into the Cox regression were gender, WIMD quintile of residence and age at diagnosis.

## Results

Between 1995 and 2020, 347 adults and 5 children were recorded in Wales with a diagnosis of Behçet’s disease. The most recent mid-year population estimate for Wales is 3.17 million [10]. Population prevalence of the disease in Wales was therefore calculated as 11.1 per 100,000 population.

Profiling was based only on the adult cases. These comprised 267 females (76.9%) and 80 males (23.1%), with years of diagnosis available for 328/347 cases (94.5%). By the end of the study period, 9 September 2021, 23 cases (6.6%) had died. Age at diagnosis was available for 328/347 cases (94.5%) with a median age of 42.0 years (range 10–78 years). WIMD data were available for 340/347 cases (97.9%), with the highest proportion of cases seen in the most deprived quintile, and the lowest proportion seen in the least deprived quintile.

Data on age at diagnosis were missing for 4/80 males (5.0%) and 15/267 females (5.6%). There was no statistically significant difference in median age at diagnosis or proportions of males and females who had died by the end of the study period. There was also no statistically significant difference between proportions of each gender living in each WIMD quintile.

There was no evidence to suggest that the age at which cases were diagnosed with Behçet’s disease had changed over time (Spearman’s Rho coefficient = 0.063, p = 0.257).

The survival analysis comparing females and males included 328/347 cases (94.5%), of which 14 females and 8 males had died. Although 23/347 cases in the study were known to have died, one female case was excluded from the survival analysis due to a date of diagnosis not being available.

**Fig. 1:**
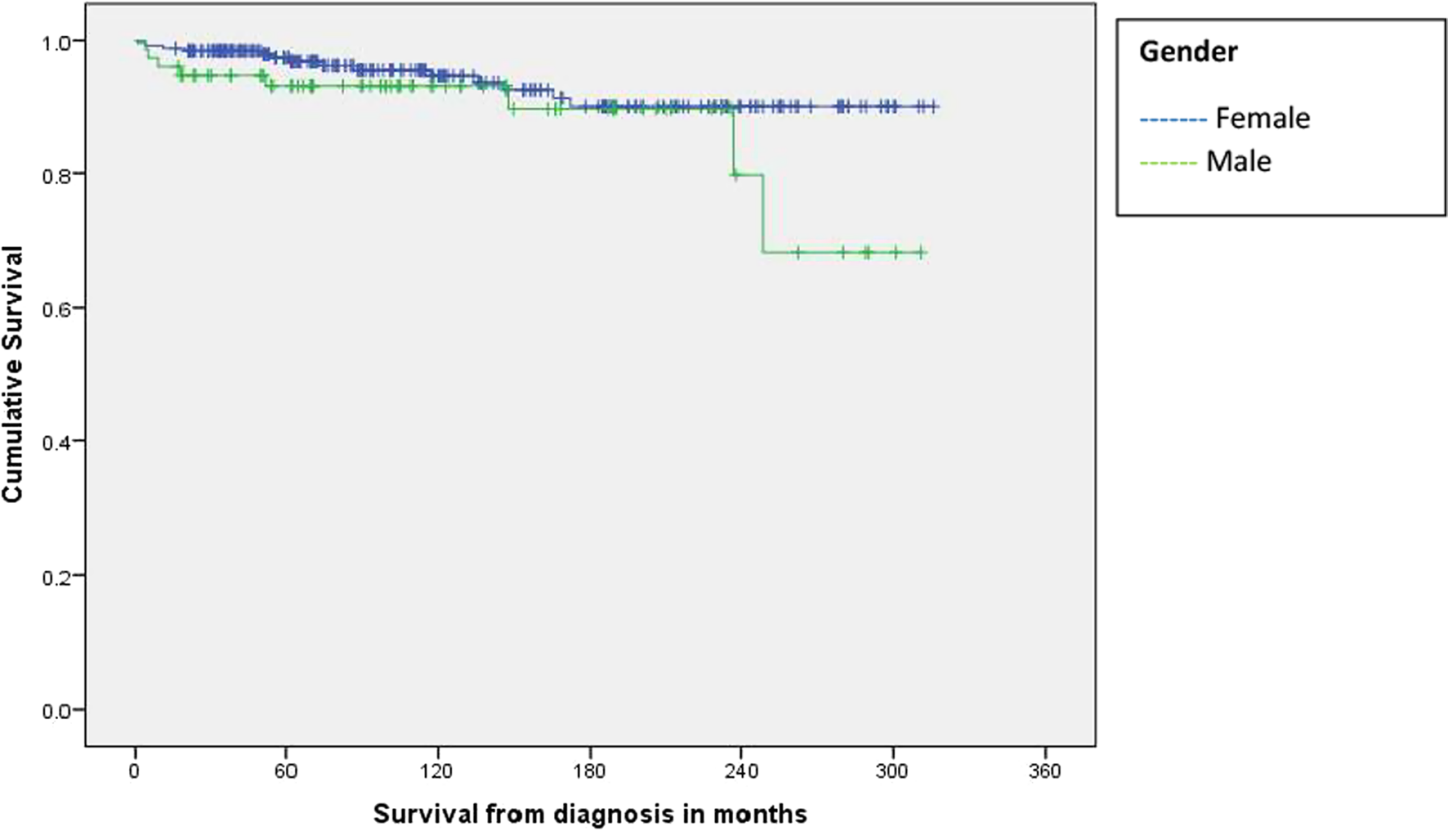
Kaplan Meier survival curve by gender

**Fig. 2:**
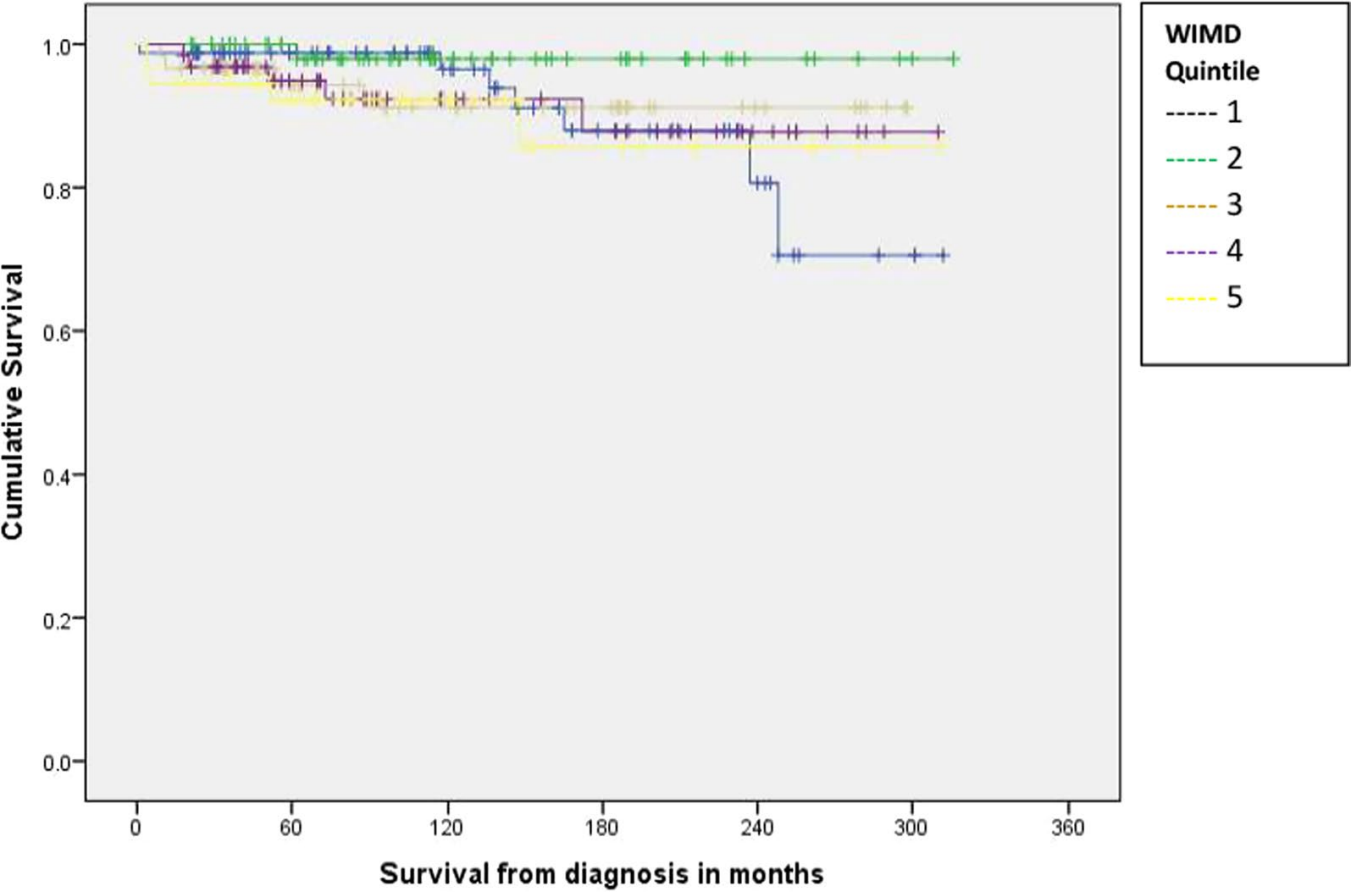
Kaplan Meier survival curve by WIMD 2019 quintile of residence

Results of the survival analysis comparing outcomes by gender, WIMD 2019 quintile and age band at diagnosis are shown in Figs. 1 and 2, and Table 1. Median survival for all cases was 101 months from diagnosis (range 1–316). There was no statistically significant difference in survival time between males and females, although the survival plot in Fig. 1 shows a drop in male survival at around 20 years from diagnosis. There was no statistically significant difference in survival between cases in each of the five WIMD 2019 quintiles (missing data for 7/347 cases), however the survival plot in Fig. 2 shows a steeper drop in survival for cases in Quintile 1 (most deprived), also at around 20 years from diagnosis, compared with all other quintiles. There was a statistically significant difference in survival time between cases diagnosed at different ages (missing data for 19/347 cases).

**Table 1:** Results of univariable and multivariable survival analyses

Factor	Survival (months)	Univariable analysis p-value (Mantel-Cox log rank test)	Multivariable analysis Exp (B) (95% CI)	Multivariable analysis p-value (Cox regression)
Gender (median, (range))		p = 0.15	Not included in final model	
Male	104.5 (4–311)			
Female	99.5 (1–315)			
WIMD 2019 quintile (median, (range))		p = 0.473		
1	119.5 (1–312)		0.60 (0.19–1.93)	p = 0.394
2	111.0 (21–316)		0.10 (0.01–0.89)	p = 0.40
3	88.0 (9–298)		0.93 (0.24–3.53)	p = 0.912
4	90.0 (18–310)		0.83 (0.24–2.89)	p = 0.772
5	101.0 (4–311)			
Age at diagnosis in years		p = 0.000	1.11 (1.08 – 1.16)	p = 0.000

Cox regression was conducted to undertake an adjusted survival analysis, comparing survival between genders when adjusting for WIMD 2019 quintile and age simultaneously. The final model in the regression identified increasing age at diagnosis as the only factor significantly and independently associated with poorer durations of survival. With each year of increasing age at diagnosis, the hazard ratio increased by 11.7%. The final step model is also given in Table 1.

## Discussion

The current population prevalence of Behçet’s disease in Wales is estimated to be 11.1 per 100,000 population. This is significantly lower than the countries of the world with the highest estimated prevalence (such as Turkey and Iran, reported as having prevalence of 380 and 68 per 100,000 population respectively) [12]. However, it is comparable with a UK prevalence estimate of 14.6 per 100,000 calculated by a 2020 cohort study [13], which noted that UK prevalence appeared to be increasing over time. A 2008 study reported wide global variation in prevalence, and acknowledged that lack of exploration of the epidemiology of the condition in many countries made the magnitude of differences in prevalence difficult to assess [12].

Our study identified that the majority (76.9%) of Behçet’s disease cases in Wales were female. Globally, studies have reported varied results, with some showing higher prevalence in males, and others showing higher prevalence in females [14]. The 2020 UK cohort study [13] also noted higher prevalence in females (63.8%).

Our findings suggest that individuals diagnosed with Behçet’s disease in Wales tend to be older than the reported average, with Behçet’s UK reporting a mean age at diagnosis of 30 years [6] and a 2020 UK cohort study describing a mean age of 35.6 years [13]. Other reports have also suggested that the majority of cases will be diagnosed while aged in their 20 s and 30 s [15]. We have reported median age in our study, however, for comparison the mean age of our cases would equate to 41.43 years. There are several plausible hypotheses for this difference. First, it is plausible that patients with milder disease may be diagnosed later than those with more severe symptoms who are more likely to have attended the specialist centres established in England in the context of the Rare Diseases Framework [16] and thus be known to Behçet’s UK. Second, there may be a longer duration of investigation prior to a definitive diagnosis for patients in Wales, as primary care physicians are unlikely to be familiar with the condition and diagnosis may require referral to multiple specialists [17].

We did not find statistically significant differences in durations of survival between males and females, or cases residing in different WIMD 2019 quintiles. These findings are perhaps surprising given that life expectancy is poorer in Wales for males than for females, and for individuals living in more deprived areas. However, Kaplan–Meier survival plots generated to investigate both these factors show steep drops in survival at around 20 years after diagnosis for both males, and individuals living in the most deprived quintile. Whilst these findings were not statistically significant, they will be kept under review. Given the low numbers of deaths recorded in the dataset, there is the possibility of a type 2 statistical error (the incorrect acceptance of the null hypothesis due to an insufficient sample size). Without access to data on causes of death, we were not able to investigate this further as part of this study. This also highlights a general difficulty in studying rare diseases, and the importance of International collaborations in order to pool data.

We note poorer durations of survival for cases diagnosed at older ages. However, as Behçet’s disease is not thought to impact on life expectancy, this finding may be a function of age itself.

The strengths of this study included national population level coverage, data collection in keeping with well-established surveillance registry practices and the triangulation of sources to ensure verification of cases.

## Limitations

- Potential for duplication due to errors in the source data. However, this was minimised by triangulation to validate cases.
- We did not include cases recorded as suspected but not validated, therefore, some genuine cases may have been missed. Some currently unvalidated cases may be validated in the future as clinical diagnoses are confirmed.
- Current data collection does not include ethnicity, Behçet’s disease severity, clinical manifestations or comorbidity data. This limited the survival analysis analytical adjustments, and collection of further variables for adults recorded in the adult rare disease registry has been noted as a consideration for future development.
- For several analyses, missing data on cases meant that not all subjects could be included; however, the impact is likely to be minimal as the number of cases with missing data was small across the cohort.
- Our survival analysis was limited in that no causes of death were recorded for cases. It was, therefore, not possible to determine whether cases had died from Behçet’s disease-related complications or other causes.
- We were unable to include calculation of a standardised mortality ratio comparing the mortality of the cohort over time with the number of expected deaths using Welsh data from age and sex specific death rates, due to the small number of deaths observed in our cohort.
- The small numbers of deaths recorded also meant that in our survival analyses, confidence intervals (as shown in Table 1) were very large and it may not have been possible to detect differences in survival between genders and people residing in different WIMD quintiles.

## Data Availability

Data included in this study is held in the Adult Rare Diseases Registry for Wales, Public Health Wales.
